# Genomic and transmission dynamics of the 2024 Marburg virus outbreak in Rwanda

**DOI:** 10.1038/s41591-024-03459-9

**Published:** 2024-12-16

**Authors:** Yvan Butera, Leon Mutesa, Edyth Parker, Raissa Muvunyi, Esperance Umumararungu, Alisen Ayitewala, Jean Pierre Musabyimana, Alhaji Olono, Placide Sesonga, Olusola Ogunsanya, Emmanuel Kabalisa, Oluwatobi Adedokun, Nelson Gahima, Laetitia Irankunda, Chantal Mutezemariya, Richard Niyonkuru, Arlene Uwituze, Ithiel Uwizera, James Kagame, Arlette Umugwaneza, John Rwabuhihi, Fidele Umwanankabandi, Valens Mbonitegeka, Edouard Ntagwabira, Etienne Kayigi, Gerard Izuwayo, Herve Murenzi, Therese Mukankwiro, Nasson Tuyiringire, Jean Marie Vianney Uwimana, Agnes Gasengayire, Reuben Sindayiheba, Glory-Ugochi Onyeugo, Merawi Aragaw, Lenny Gitundu, Radjabu Bigirimana, Mosoka Fallah, Adaora Ejikeme, Senga Sembuche, Alice Kabanda, Jean Claude Mugisha, Emmanuel Edwar Siddig Francis, Pierre Gashema, Jerome Ndayisenga, Alexis Rugamba, Faustin Kanyabwisha, Gad Murenzi, Anise Happi, Jean Claude Semuto Ngabonziza, Misbah Gashegu, Ayman Ahmed, Noella Bigirimana, Edson Rwagasore, Muhammed Semakula, Jean Paul Rwabihama, Clarisse Musanabaganwa, Eric Seruyange, Menelas Nkeshimana, Theogene Twagirumugabe, David Turatsinze, Eric Remera, Noel Gahamanyi, Sofonias Kifle Tessema, Isabelle Mukagatare, Sabin Nsanzimana, Christian Happi, Claude Mambo Muvunyi

**Affiliations:** 1 Rwanda Joint Task Force for Marburg Virus Disease Outbreak, Ministry of Health, Rwanda Biomedical Centre, Kigali, Rwanda; 2 Ministry of Health, Kigali, Rwanda; 3 Center for Human Genetics, College of Medicine and Health Sciences, University of Rwanda, Kigali, Rwanda; 4 Africa Centre for Disease Control and Prevention, Addis Ababa, Ethiopia; 5 African Center of Excellence for Genomics of Infectious Diseases, Redeemer’s University, Ede, Nigeria; 6 Department of Immunology and Microbiology, The Scripps Research Institute, La Jolla, CA USA; 7 Rwanda Biomedical Centre, Kigali, Rwanda; 8 Department of Internal Medicine, College of Medicine and Health Sciences, University of Rwanda, Kigali, Rwanda; 9 Rwanda Military Referral and Teaching Hospital, Kigali, Rwanda; 10 Butare University Teaching Hospital, Huye, Rwanda; 11 Kigali University Teaching Hospital, Kigali, Rwanda; 12 Department of Immunology and Infectious Diseases, Harvard T H Chan School of Public Health, Boston, MA USA

**Keywords:** Viral infection, Phylogenetics

## Abstract

The ongoing outbreak of Marburg virus disease in Rwanda marks the third largest historically, although it has shown the lowest fatality rate. Genomic analysis of samples from 18 cases identified a lineage with limited internal diversity, closely related to a 2014 Ugandan case. Our findings suggest that the Rwandan lineage diverged decades ago from a common ancestor shared with diversity sampled from bats in Uganda. Our genomic data reveal limited genetic variation, consistent with a single zoonotic transmission event and limited human-to-human transmission. Investigations including contact tracing, clinical assessments, sequencing and serology, linked the index case to a mining cave inhabited by *Rousettus aegyptiacus*. Serology tests identified three individuals seropositive for immunoglobulin G and immunoglobulin M, further supporting the zoonotic origin of the outbreak through human–animal interactions.

## Abstract (web summary)

Genomic analyses from the ongoing outbreak of Marburg virus disease in Rwanda point to a single zoonotic origin for the outbreak.

## Main

Since its discovery in 1967, Marburg virus disease (MVD) has emerged as a significant global health threat, resulting in several outbreaks characterized by alarmingly high case fatality rates ranging from 22% to 90% (ref. ^1^). The first reported instances of MVD occurred in simultaneous outbreaks in Marburg and Frankfurt, Germany, as well as in Belgrade, Yugoslavia (now Serbia)^2^. To date, there have been 19 recorded outbreaks, 563 confirmed cases and 428 deaths worldwide^3^. On 27 September 2024, Rwanda reported its first ever cases of MVD^4^. The ongoing outbreak in Rwanda has confirmed 66 cases, with a case fatality rate of approximately 23% (ref. ^4^).

MVD has two distinct variants, Marburg virus (MARV) and Ravn virus, with MARV being the more prevalent and responsible for the majority of sporadic MVD outbreaks worldwide. MARV is a negative-sense single-stranded RNA virus from the Filoviridae family, similar to Ebola and possesses inverse-complementary 3′ and 5′ termini^3^. MVD is predominantly a zoonosis, transmitting to humans from either the bat natural reservoir or through intermediate hosts, including nonhuman primates. The mechanisms by which bats regulate viral replication and maintain complex immunity in contrast to humans remain unclear because of a lack of bat-specific research tools necessary for comparative studies, such as antibodies for flow cytometry, genomics and transcriptomics^1,2^. Studies confirm that MARV’s natural reservoir is the Egyptian fruit bat (*Rousettus aegyptiacus*)^5^. The virus can cause viral hemorrhagic fever (VHF) in both humans and primates. However, the precise transmission dynamics between the natural reservoir and humans or primates are insufficiently understood, although exposure to contaminated excretions from fruit bats is likely a contributing factor^5^. The virus is transmitted from human to human, particularly through direct contact with blood, saliva, semen and other body fluids from infected individuals^6^.

The Marburg viral genome consists of 19,114 nucleotides encoding for seven proteins, including glycoprotein (GP), nucleoprotein (NP) virion protein 30 (VP30), VP35, VP24, VP40 and large viral polymerase^3^. Genomic characterization of MARV strains has revealed substantial genetic diversity across its global phylogeny, but has limited genomic variation in outbreaks^7^.

Sequencing of the Marburg genome has provided deeper insights into viral evolution and identified critical mutation hotspots during adaptation to new hosts, particularly in the VP40 protein and the NP–VP35 intergenic region^8^. These mutations can influence the virus’s ability to evade the immune system, therefore enhancing its pathogenicity.

In this study, we used a near real-time genomics sequencing approach to characterize the Marburg virus in blood samples obtained from patients in the ongoing outbreak in Rwanda. Our aim was to use the genomic sequencing data to identify and characterize the virus and understand its evolution and transmission dynamics during the current outbreak. This research underscores the importance of continuous genomic surveillance, which not only facilitates outbreak tracking, but also informs public health strategies for effectively controlling this deadly pathogen.

**Fig. 1:**
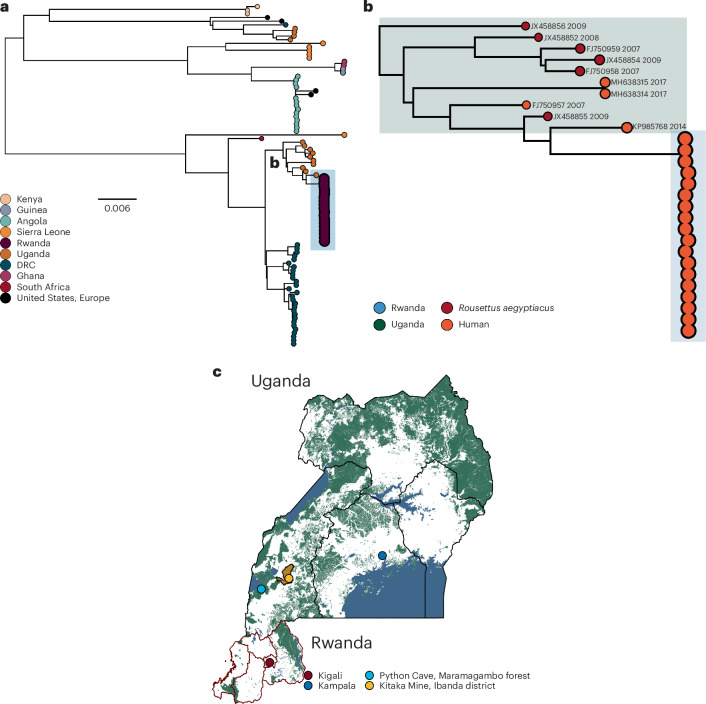
Phylogenetic analyses of the Rwanda MARV outbreak. **a**, Maximum likelihood phylogeny of the global MARV dataset. Tips are annotated by country of isolation. This study’s tips are shown relatively enlarged. **b**, The Rwandan outbreak clade nested in diversity sampled from sporadic human cases and fruit bats, respectively, in Uganda. **c**, Map of Uganda and Rwanda. The city for patients in this study (Kigali) is annotated in dark red. Python Cave and Kitaka Mine in Ibanda district in southwestern Uganda are annotated in light blue and yellow, respectively. DRC, Democratic Republic of the Congo.

**Extended Data Fig. 1:**
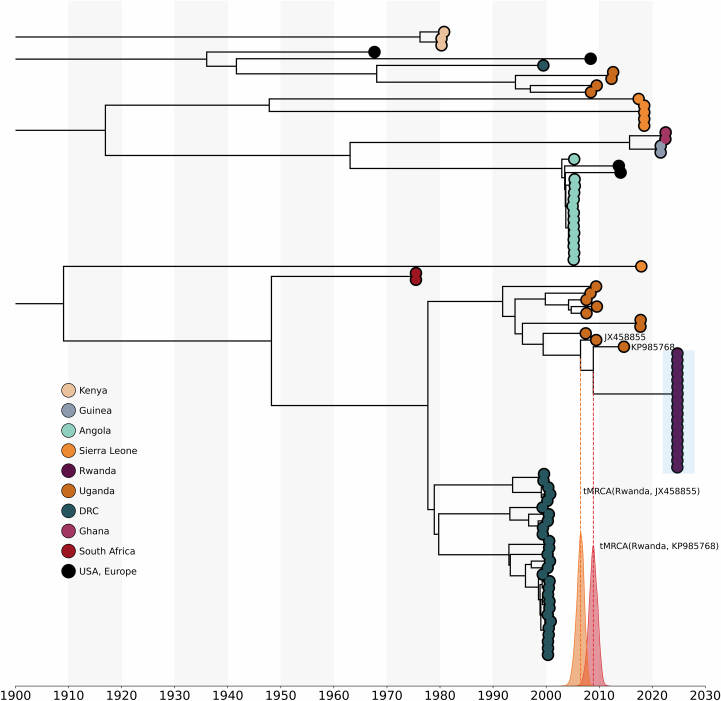
Time resolved maximum clade credibility tree of the global MARV and relationship with the Rwanda outbreak lineage. Time resolved maximum clade credibility tree of global MARV dataset, with closest human (KP985768) and zoonotic (JX458855) sequence to the Rwandan outbreak lineage annotated. The tMRCAs of the Rwandan lineage and KP985768 and JX458855 respectively are highlighted in red and orange.

Rwanda effectively contained the spread of MARV from further reaching the broader community by prompt isolation of positive cases and treatment with monoclonal antibodies and antivirals. Over the span of the outbreak, the case fatality rate was approximately 23%. We generated genomes from 18 early cases to investigate the zoonotic origin and transmission dynamics of MARV in the early epidemic. Our phylogenetic analyses support that the outbreak resulted from a single zoonotic transmission event with limited human-to-human transmission rather than multiple independent zoonotic transmission events (Fig. 1a,b). Multiple independent zoonotic transmissions would have resulted in a set of more diverged sequences introduced from the genetically diverse viral population in the reservoir^9^. The outbreak lineage is most closely related to a sequence sampled in Kampala, Uganda in September 2014 from a healthcare worker, with no secondary cases observed (KP985768). The source of the zoonotic transmission in 2014 was never identified^10^. However, the Rwanda–Uganda sister lineages are significantly diverged from one another, separated by 82 nucleotide substitutions (Fig. 1a,b). In Bayesian phylogenetic reconstructions, we estimate that the two lineages diverged from a common ancestor that circulated in the animal reservoir in November 2008 (95% highest posterior density: May 2007 and June 2010) (Extended Data Fig. 1).

The outbreak lineage in Rwanda is nested in a larger clade that includes diversity sampled from bats in southwestern Uganda from 2007 onwards, along with two human cases identified in Uganda in 2017 (Fig. 1a,b). The closest bat virus (JX458855) was sampled from a juvenile Egyptian fruit bat (*R. aegyptiacus*) in 2009 from Python Cave in Queen Elizabeth National Park, a popular tourist attraction in southwestern Uganda (Fig. 1b)^5^. The lineage in Rwanda is significantly diverged from JX458855, sharing a common ancestor that likely circulated in the reservoir as early as June 2006 (95% highest posterior density: December 2004 to November 2007) (Extended Data Fig. 1). *R. aegyptiacus* are a cave-dwelling species, with spillover events and limited outbreaks frequently associated with mining activities. This includes outbreaks and sporadic cases linked to Python cave and the Kitaka mine that is only 50 km away in 2007 and 2008, both of which host *R. aegyptiacus* colonies of more than 50,000 bats^5,9,11^. The divergent relationship of the Rwandan Marburg virus outbreak lineage indicates that the virus's dispersal through host networks involved larger scale animal movement over a long time, rather than a direct ancestral connection to the *R. aegyptiacus* colony in southwestern Uganda.

**Fig. 2:**
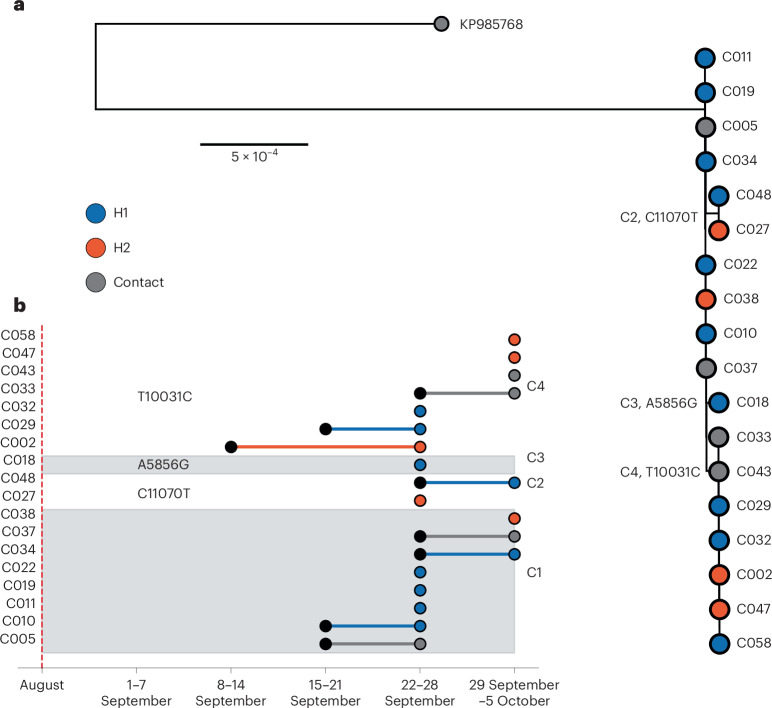
Time-dependent SNPS reconstruction of the MARV during the 2024 Rwanda outbreak. **a**, Maximum likelihood phylogeny of the outbreak clade, with the Kampala outgroup. Sequences are colored by the associated hospital. SNPs reconstructed relative to the common ancestor are annotated in the text, as are the clusters defined by the presence of SNP. The coordinates are relative to NC_001608. **b**, Epidemiological timeline of the sampled sequences in **a**, as annotated on the *y* axis. The first black marker, if present, indicates the week of symptom onset if distinct from week of sampling, with the second marker indicating the week of sampling. Sequences are colored by their associated hospital (H1, H2), and partitioned by SNP presence into clusters, as annotated in text. The red dashed line indicates the contact of the index case admitted to H1.

The index patient’s close relative was admitted to hospital (H) 1 at the end of August 2024 but succumbed to the illness before a definitive diagnosis of MVD. It is highly probable that this patient infected healthcare workers at H1, where MVD was subsequently confirmed at the end of September 2024. Between 22 and 28 September, some 26 cases were reported in healthcare workers at H1 and H2, respectively. The limited genetic variation among the outbreak sequences was consistent with a very recent common ancestor, the short sampling period and previous observations from the Angola MARV outbreak (Fig. 2a)^11^. A cluster of eight identical sequences was sampled, spanning the full sampling period (cluster C1 in Fig. 2a,b). The majority of the C1 sequences were isolated from healthcare workers at H1 between 22 and 28 September, with reported symptom onset as early as mid-September (Fig. 2b). A C1 sequence was sampled from a healthcare worker at H2 at the end of September, suggesting infection from an unsampled C1 case in H2 or dissemination between the two healthcare facilities. There are recorded cases of healthcare workers moving between healthcare settings H1 and H2. This is further supported by cluster C2, consisting of two healthcare workers from H1 and H2, respectively, that share the synonymous single nucleotide polymorphism (SNP) C11070T relative to C1. Both C2 cases report symptom onset and were sampled in the later weeks of the epidemic. There are a further seven identical sequences (C4) sampled between H1 and H2 across the full sampling period that are one synonymous SNP (T10031C) away from C1. C002, a healthcare worker employed at both H1 and H2, is believed to have acquired the infection at H1. This individual subsequently became the index case at H2, leading to a nosocomial cluster at H2 and infections at H1. Overall, there were only three synonymous SNPs observed in the outbreak clade: A5856G (*n* = 1 sequence), T10031C (*n* = 7) and C11070T (*n* = 2). A5856G and T10031C as well as C11070T are outside the coding DNA sequence of the *GP* and *VP24* genes, respectively. There is, therefore, no evidence that post-emergence substitutions enhanced human transmission.

**Extended Data Fig. 2:**
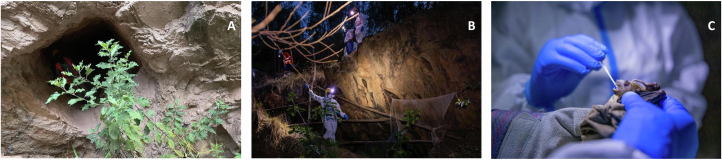
Mining cave, bat trapping and sampling for MARV in Rwanda. **a**) *Mining cave entrance with human activity;* **b**) *Trapping fruit bats with net*; **c**) *Sampling bats for Marburg virus*.

We conducted an exhaustive epidemiological investigation to identify the index case, including reviews of clinical records, travel history, antibody serology testing results and contact tracing information. In this investigation, we identified a group of individuals linked to a mining cave and screened them for MARV. All individuals were polymerase chain reaction (PCR) negative, but serological testing revealed that three individuals had antibodies indicating previous exposure to Marburg virus. We found that the probable index case of this group was a man in his 20s with an occupational exposure to fruit bats (*R. aegyptiacus*) in a mining cave environment. The individual had the earliest symptoms, which were highly consisted with the classic clinical presentation of MVD. In addition, serological testing of three contacts of the index case had MARV antibodies, further supporting epidemiological tracing of the index case. However, no sequence data were obtained from these cases. In response to these findings, the Rwanda Ministry of Health through a multisectoral collaboration initiated a surveillance investigation e aimed at screening fruit bats, primarily cave dwellers, for Marburg virus (Extended Data Fig. 2a–c).

The Rwanda MVD outbreak primarily involved limited onward human-to-human transmission between cases and healthcare personnel, as is often observed with VHF outbreaks closely associated with nosocomial and occupational infections^12^. This underscores the ongoing need to enhance healthcare personnel’s knowledge and attitudes regarding VHF case management. In our phylogenetic analyses, we observed that the early outbreak sequences represented limited genomic variation, indicating that the outbreak originated from a single zoonotic transmission event. The Rwandan lineage shared a common ancestor with sequences originating from diversity sampled in bats in southwestern Uganda, thought the lineages had diverged over decades in the animal reservoir. It is likely that enhanced zoonotic surveillance will reveal many unsampled intermediates that can clarify the ecological pathways of transmission between bat colonies in Uganda and Rwanda. Previous research indicates that bats can harbor MARV for extended periods, with active mining areas in Rwanda providing ideal habitats for bats and increasing the likelihood of zoonotic transmission^13^.

We observed three synonymous SNPs outside the coding regions of the *GP* and *VP24* genes, respectively. Studies have shown that the Marburg virus *VP24* protein interacts with the NP and other cellular membranes, facilitating the release of new virions from infected cells^14^. In addition, research highlights that MARV *VP24* directly interacts with the human and bat Keap1 proteins, which modulate antioxidant responses to support viral replication, a strategy likely critical for viral persistence and host adaptation^15^. Genomic sequencing of MARV in Rwanda has yielded critical insights into viral circulation dynamics. However, as these SNPs fall outside coding regions, it is unlikely that they functionally contribute to enhanced viral fitness. Our results should be interpreted in the context of our smaller sample size, which only covers the first 2 weeks of the outbreak.

This research underscores the importance of continuous genomic surveillance that not only facilitates outbreak tracking, but also informs public health strategies for effectively controlling this deadly pathogen. In addition, previous research indicates that bats can harbor MARV for extended periods, with active mining areas in Rwanda providing ideal habitats for bats and increasing the likelihood of zoonotic transmission. Furthermore, Rwanda’s location in a region of consistent turbulent outbreaks, including Ebola, Rift Valley fever, COVID-19, dengue and Marburg, underscores the importance of regional and international collaboration to enhance outbreak preparedness and responses for global health security.

Our study provides valuable insights into the evolutionary dynamics of MARV. However, our findings should be interpreted in the context of our limited sample. Our sample did not encompass all positive cases from the outbreak because of the rapid timeline required for an urgent response. In addition, the availability of historical genomic data for comparison was limited, including MARV samples from animals, because gaps in sequencing capacity restricted the inclusion of sequences from recent outbreaks.

## Methods

### Ethics declaration

The study was approved by the Rwanda National Ethics Committee (Federalwide Assurance Assurance no. 00001973 IRB 00001497 of IORG0001100-Protocol approval notice: no. 121/RNEC/2024). All necessary patient/participant consent has been obtained and the appropriate institutional forms have been archived. Patient/participant/sample identifiers included were not known to anyone (for example, hospital staff, patients or participants themselves) outside the research group so cannot be used to identify individuals. Under the circumstances of the emergency of the outbreak verbal consent was obtained.

### Patient sample collection

We obtained whole blood samples from suspected patients presented with clinical symptoms (high fever, severe headaches, muscle aches, fatigue, nausea, vomiting and diarrhea) of MDV. Sample testing was performed at Rwanda Biomedical Centre/National Reference Laboratory, whereby samples were kept in a cold chain before plasma separation and analysis.

### Nucleic acid extraction

Viral RNA was extracted from 140 µl of plasma using the QIAamp Viral RNA Mini Kit (Qiagen), following the manufacturer’s instructions adapted to the in-house standard operating procedures with an elution volume of 60 µl. The extracted RNA was quantified using a Qubit fluorometer, and the samples were stored at −80 °C until further analysis.

### PCR with reverse transcription and genomic sequencing

For the detection and amplification of the target regions of the MVD, we used the use of RealStar Filovirus Screen RT–PCR Kit 1.0 (Altona Diagnostics), which is based on real-time PCR technology, for the qualitative detection and differentiation of Ebola and Marburg virus specific RNA in human EDTA plasma. Quantitative PCR with reverse transcription was conducted following manufacturer’s user guide and performed on CFX96 BIORAD machine. The amplification followed an initial denaturation at 95 °C for 5 min, followed by 40 cycles of denaturation at 95 °C for 30 s, annealing at 55 °C for 30 s and extension at 72 °C for 1 min, concluding with a final extension at 72 °C for 5 min. The amplified products were analyzed via 2% agarose gel electrophoresis to confirm successful amplification and appropriate product size.

Following amplification, library construction was performed on 30 samples using the Illumina RNA Prep with Enrichment (L) Tagmentation workflow and the lllumina Viral Surveillance Panel v2 kit. Total RNA extracts were converted to complementary DNA, tagmented and thereafter amplified. The genomic region of interest was captured using a hybrid capture method. Probes were isolated using magnetic pulldown and selectively enriched for the desired regions. Enriched libraries were quantified using double strand DNA high-sensitivity assay and Qubit fluorometer. Libraries were thereafter denatured and normalized at a final loading concentration of 0.8 pM. Paired-end sequencing was performed using a NextSeq 550 with a 300-cycle mid-output cartridge, with the sequencing depth aimed at a minimum coverage of 100× to ensure robust variant detection.

### Bioinformatics analysis

#### Genome assembly

We used a reference-based genome assembly pipeline to analyze the sequencing data generated using the Illumina Viral Surveillance Panel v2 kit (described above). This workflow integrates several steps including quality control, host genome filtering and viral genome assembly to accurately reconstruct viral sequences from targeted sequencing. To ensure that only high-quality data were processed downstream, raw sequencing reads were first processed with fastp v.0.23.24 (ref. ^16^) for trimming adapters and filtering out low-quality bases. Following this, human host genome sequences were filtered out by aligning the cleaned reads to the human genome (hg38) using Bowtie2 v.2.5.4 (ref. ^17^). Unmapped reads, presumed to be of viral origin, were retained for downstream analysis. These de-hosted reads were then aligned to a Marburg virus reference genome (NC_001608.3) using Minimap2 v.2.28 (ref. ^18^), the sequences had between 70% and 97% genomic coverage. We manually masked two mutations in respective consensus sequences that were adjacent to potential misalignment.

#### Variant calling and consensus generation

Variant calling was performed using both ivar v.1.4.3 (ref. ^19^) and LoFreq v.2.1.5 (ref. ^20^) for comparison. A depth coverage threshold of 50 reads per nucleotide position was applied to ensure robust and reliable variant detection. This threshold was also applied to the generation of consensus sequences which was also done using ivar v.1.4.3.

#### Phylogenetic analyses

We combined our 18 higher quality sequences (coverage ≥70%) with all publicly available MARV sequences available on GenBank (*N* = 81). To root the tree, we included wight Ravn virus sequences included as an outgroup, which were pruned from the tree and excluded in all subsequent analyses. We aligned the sequences using Mafft v.7.52 (ref. ^21^) and reconstructed a phylogenetic tree using IQTree v.2.2.5 (ref. ^22^) under modelfinder plus^23^, with ultrafast bootstrapping^24^. We performed the initial ancestral state reconstruction using Treetime v.0.9.3 (ref. ^25^).

#### Bayesian phylogenetic reconstruction

**Extended Data Fig. 3:**
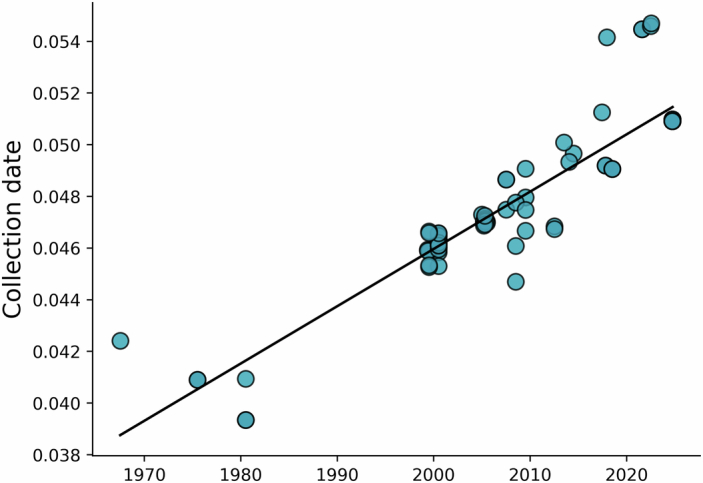
Temporal regression of the global Marburg phylogeny in Extended Data Fig. 1. Each point indicates a sequence’s divergence from the root, plotted against sampling time. The regression indicates sufficient temporal signal to resolve a time-resolved phylogeny.

The current genomic data do not provide sufficient temporal signal to estimate the rate of evolution in the outbreak clade. However, we were not interested in estimating the time to the most recent common ancestor of the outbreak clade, because determining the emergence timing of the outbreak clade will more likely depend on the epidemiological and contact history of the index case. We wanted to estimate time to the most recent common ancestor of the outbreak clade and its closest human and zoonotic outgroups to better understand its potential zoonotic emergence pathway. We therefore reconstructed the time-resolved phylogeny under a fixed local clock using the BEAST software package. We assumed a fixed value of 1 × 10^−3^ substitutions per site per year as a plausible rate of evolution for an RNA virus in the early stage of an outbreak for the defined outbreak clade. We allowed the remainder of the tree to evolve under a lognormal prior centered on 5 × 10^−4^ (ref. ^11^). We used a two-phase coalescent model: the tree from the most recent common ancestor (Rwandan lineage) onward was modeled with an exponential growth model, with the earlier phase modeled as a constant-population size coalescent model. We ran two independent chains of 100 million states to ensure convergence, discarding the initial 10% of each chain as burn-in. The chains were then combined with LogCombiner. For all subsequent analyses, we assessed convergence using Tracer, and constructed a maximum clade credibility tree in TreeAnnotator v.1.10 (ref. ^26^). A plot of the temporal regression is given in Extended Data Fig. 3.

### Reporting summary

Further information on research design is available in the Nature Portfolio Reporting Summary linked to this article.

## Online content

Any methods, additional references, Nature Portfolio reporting summaries, source data, extended data, supplementary information, acknowledgements, peer review information; details of author contributions and competing interests; and statements of data and code availability are available at 10.1038/s41591-024-03459-9.

## Supplementary information

Reporting Summary

## Data Availability

All the sequences are available on National Center for Biotechnology Information GenBank under accession numbers PQ552725–PQ552742 (https://linkmix.co/31343096). All analyses code and data are available at https://github.com/rbc-bioinformatics/vsp-genomic-assembly-pipeline/ and https://github.com/EdythParker/MARV_transmission_dyanmics_in_Rwanda_outbreak, excluding shapefiles, which are available on request owing to size. All shapes files were obtained from the Food and Agriculture Organization geoNetwork. All pipelines are publicly available, and any issues or inquiries can be addressed through the respective GitHub pages, where they will be promptly attended to within 24 hours. For assistance with the genome assembly pipeline contact bfx@rbc.gov.rw, and for phylogenetic analyses contact edythp@run.edu.ng.

## Code availability

All code to run the analyses and make figures are available via GitHub at https://github.com/EdythParker/MARV_transmission_dyanmics_in_Rwanda_outbreak. Code used for genome assembly is available via GitHub at https://github.com/rbc-bioinformatics/vsp-genomic-assembly-pipeline/.
